# Vitamin D deficiency is associated with poorer satisfaction with diabetes-related treatment and quality of life in patients with type 2 diabetes: a cross-sectional study

**DOI:** 10.1186/s12955-018-0873-3

**Published:** 2018-03-12

**Authors:** Nuria Alcubierre, Esmeralda Castelblanco, Montserrat Martínez-Alonso, Minerva Granado-Casas, Aureli Esquerda, Alicia Traveset, Dolores Martinez-Gonzalez, Josep Franch-Nadal, Didac Mauricio

**Affiliations:** 1 Department of Nutrition and Dietetics, Avantmèdic, 25008 Lleida, Spain; 2 Department of Endocrinology and Nutrition, Health Sciences Research Institute Germans Trias i Pujol, 08916 Badalona, Spain; 3 Centre for Biomedical Research on Diabetes and Associated Metabolic Diseases (CIBERDEM), Instituto de Salud Carlos III, Badalona, Spain; 4 Biostatistics Unit, Biomedical Research Institute of Lleida (IRBLleida), University of Lleida, 25198 Lleida, Spain; 5 Biomedical Research Institute of Lleida, University of Lleida, 25198 Lleida, Spain; 6 Department of Laboratory Medicine, University Hospital Arnau de Vilanova, 25198 Lleida, Spain; 7 Department of Ophthalmology, University Hospital Arnau de Vilanova, 25198 Lleida, Spain; 8 Unitat de Suport a la Recerca de Barcelona, Institut Universitari d’Investigació en Atenció Primària Jordi Gol (IDIAP Jordi Gol), 08007 Barcelona, Spain; 9 Departament of Medicine, University of Barcelona, Barcelona, Spain

**Keywords:** Type 2 diabetes mellitus, Vitamin D, Health-related quality of life, Treatment satisfaction, Diabetic retinopathy

## Abstract

**Background:**

In this cross-sectional study, we assessed the possible association of vitamin D deficiency with self-reported treatment satisfaction and health-related quality of life in patients with type 2 diabetes.

**Methods:**

We performed a sub-analysis of a previous study and included a total of 292 type 2 diabetic patients. We evaluated treatment satisfaction and health-related quality of life through specific tools: the Diabetes Treatment Satisfaction Questionnaire and the Audit of Diabetes-Dependent Quality of Life. Vitamin D deficiency was defined as 25 (OH) D serum levels < 15 ng/mL.

**Results:**

Multivariable linear regression models were used to estimate the relationship of vitamin D deficiency with both outcomes once adjusted for self-reported patient characteristics. Vitamin D deficiency was significantly associated with the final score of the Diabetes Treatment Satisfaction Questionnaire and the single “diabetes-specific quality of life” dimension of the Audit of Diabetes-Dependent Quality of Life (*p* = 0.0198 and *p* = 0.0070, respectively). However, lower concentrations of 25-OH vitamin D were not associated with the overall quality of life score or the perceived frequency of hyperglycaemia and hypoglycaemia.

**Conclusions:**

Our study shows the association between vitamin D deficiency and both the self-reported diabetes treatment satisfaction and the diabetes-specific quality of life in patients with type 2 diabetes.

## Background

Type 2 diabetes mellitus (T2DM) has a negative impact on the quality of life of the people who suffer from it. T2DM involves the physical and emotional overload of a disease that “has no cure”, that requires life-long treatment and that has therapeutic measures that include the introduction of lifestyle changes and pharmacological treatment, often with multiple drugs [1]. The preservation of health-related quality of life (HRQoL) and the optimisation of satisfaction with the treatment administered stand as two important objectives for the patient with T2DM [2, 3].

Although traditionally vitamin D has been associated with calcium-phosphate metabolism, recent epidemiological studies show the relation between hypovitaminosis D and several different diseases or conditions, such as diabetes, cancer, autoimmune disorders, and infectious, respiratory, or cardiovascular diseases [4]. This diverse physiological burden can be expected as there are vitamin D receptors in different tissues and the activation of these receptors not only induces the modification of the expression of those genes involved in mineral homeostasis and bone remodelling but also induces the expression of more than 200 genes involved in different cellular pathways that affect mechanisms such as immunomodulation, the control of hormone secretion, inhibition of cell growth, and induction of cell differentiation. With respect to diabetes, vitamin D is involved in the secretion and action of insulin and may influence chronic low-grade inflammation and angiogenesis [5, 6]. There is an association of vitamin D insufficiency and increased fat infiltration in skeletal muscle, independently of body mass, that might contribute to a decreased insulin action [7]. In addition, there is increasing evidence of the possible role that severe vitamin D deficiency plays as a modifiable risk factor for mortality, specifically of both all-cause and cardiovascular mortality in patients with T2DM, and of its association with the presence and severity of multiple comorbidities [8–10].

Recently, the possible impact of vitamin D deficiency on HRQoL and other aspects that are of great importance for the patient (biophysiological, emotional and social considerations) have been studied in subjects with various conditions (osteoarthritis, osteoporosis, inflammatory bowel disease and chronic kidney disease) and in healthy populations [11–19]. Nevertheless, there are still very few studies that have analysed the impact of vitamin D deficiency on the HRQoL of these patients and none of these studies has assessed its effects on the satisfaction with treatment [16–20]. Concerning diabetes, a recent cross-sectional study in non-vitamin D deficient Dutch subjects, with fair metabolic control of their T2DM, found no association between vitamin D levels and HRQoL [21]. Similarly, in a randomised, double-blind, placebo-controlled trial, the same researchers showed that there was no effect of vitamin D supplementation (50,000 IU for six months) on self-reported HRQoL, which was assessed using the Short Form 36 Health Survey, in patients with similar characteristics [22]. Meanwhile, Mager et al. analysed the impact of six months of different doses of vitamin D3 supplementation (2000 IU/daily or 40,000 IU/monthly), administered to Canadian adults with diabetes mellitus (more than 95% with T2DM) and other chronic diseases, on the following primary outcomes: vitamin D status, bone health and Fibroblast Growth Factor-23, and on the following secondary outcome: HRQoL. The results of this open-label randomised clinical trial did not show any significant improvement in bone mineral density or HRQoL [23]. It must be noted that all the studies on diabetes that are mentioned above excluded subjects with vitamin D deficiency. In contrast, the results of a recent sub-analysis of the Comprehensive Dialysis Study, with a well-characterised cohort of incident dialysis patients (60.4% with diabetes), did find an association between 25-OH vitamin D deficiency (< 15 ng/ml) and poorer self-reported mental health and physical activity [10]. Thereby, the status of vitamin D is related to unfavourable outcomes of disease and complications that affect the quality of life of patients. In the current study, we hypothesised that, in patients with T2DM, vitamin D deficiency is associated with lower levels of satisfaction with their treatment and poorer HRQoL.

## Methods

### Study design, participants and study procedures

The current study is the result of a sub-analysis of a previous cross-sectional study conducted by our research group. The primary objective of the current study was to evaluate the impact of retinopathy on the quality of life and treatment satisfaction in patients with T2DM without any other advanced complications of diabetes [2]. From a total sample of 297 participants, aged 40–75 years, we included a final number of 292 subjects. Five subjects were excluded from the initial study because they were receiving vitamin D supplementation during the six months prior to recruitment. Concentrations of 25-OH vitamin D were measured using a chemiluminescent microparticle immunoassay in an Architect i2000SR analyser (Abbott Diagnostics, Lake Forest, IL, USA), with an intra-assay and inter-assay variability of 2.3% and 6.2%, respectively. Vitamin D deficiency was defined based on the cut-off point of serum vitamin D concentrations below 15 ng/mL (37.4 nmol/L). This cut-off concentration was found to define the vitamin D deficiency threshold below which there was a higher frequency of diabetic retinopathy in the subjects of a previous study [9]. Additionally, all three previous studies addressing the issue of the association of vitamin D and quality of life in diabetic patients used this concentration as the limit to define vitamin D deficiency [21–23]. A patient was classified as having dyslipidaemia and hypertension when she/he received medication for these conditions. The characteristics of the study population and the detailed procedures of the study were described and reported in detail in a previous publication [2]. The study was approved by the Ethics Committee of our institution in accordance with the Declaration of Helsinki. All study participants provided signed informed consent.

### Patient-reported outcomes

To assess HRQoL, we used the latest version of the specific quality of life questionnaire for diabetic patients, the Audit of Diabetes-Dependent Quality of Life (ADDQoL-19), which is designed to assess the patient’s personal perspective on the impact of diabetes and its treatment on quality of life [24]. The first two items are general and scored separately: the first measures current quality of life, from − 3 (extremely bad) to + 3 (excellent), while the second evaluates the overall impact of diabetes on quality of life, from − 3 (maximum negative impact of diabetes) to + 1 (maximum positive impact of diabetes). The individual items consist of questions on 19 specific dimensions of life (such as social and affective life). The ADDQoL allowed us to calculate a final weighted score, the average weighted impact, which ranged from − 9 (maximum negative impact of diabetes) to + 3 (maximum positive impact of diabetes) and weighted the effects of diabetes and its treatment on the quality of life of the participants [24]. The ADDQoL has previously been validated in our country in patients with T2DM [25, 26].

Similarly, to measure the patient’s satisfaction with her/his treatment, we chose the Diabetes Treatment Satisfaction Questionnaire–status version (DTSQ-s), which is designed to assess the degree of satisfaction of diabetic patients with the treatment they receive [27]. This instrument has also been validated for the Spanish population [28]. It consists of eight questions, two of which are scored separately (perception of the frequency of hyperglycaemia and hypoglycaemia). All the items have seven possible answers, ranging from 0 to 6. The degree of overall satisfaction (final score) is expressed by a global score of 0 to 36, with higher values expressing greater degrees of satisfaction with treatment [27]. The use of the DTSQ-s has been widely recommended by the World Health Organisation and by the International Diabetes Federation as a valid instrument that allows for the accurate measurement of patient satisfaction with their treatment in patients with types 1 or 2 diabetes [29].

### Statistical analysis

Non-normally distributed quantitative variables were described by median values (interquartile range) and were compared between vitamin D deficiency groups using the Mann-Whitney test. Normally distributed quantitative variables were described by mean values (standard deviation) and were compared between both groups using the Student’s t-test. Qualitative variables were summarised as frequencies (percentages) and were compared using the chi-squared test. The statistical analysis included the estimation of univariate and multivariable linear regression models for the variability of the overall mean score of quality of life and the single item assessments of “present quality of life” and “diabetes-specific quality of life” provided by the ADDQoL. The treatment satisfaction score and the hyperglycaemia and hypoglycaemia frequencies were also fitted to univariate and multivariable linear regression models to assess their relationship with vitamin D before and after adjusting by significantly related patient characteristics. We assessed the significant contribution (by likelihood ratio test) or confounding effect (detected by changes in coefficients over a 15%) for all the patients’ characteristics collected and reported in Table 1. Interactions found in the previous study were included in the models [2]. A robust quantile regression for the median score was performed in case of deviations from the linear model assumption of normally distributed residuals as assessed by normal probability plot. The estimated post-host statistical power was 100% and 99.8% for models of diabetes-specific quality of life and Diabetes Treatment Satisfaction Questionnaire score, given their coefficients of determination. A significance level of 0.05 and the statistical software R were used.

**Table 1:** Demographic and clinical characteristics of the study subjects

	All patients(*n* = 292)	No Vitamin D deficiency (*n* = 192)	Vitamin D deficiency (*n* = 100)	*p*-value
Vitamin D (ng/mL)	18.4 [13.1;25.0]	22.7 [18.7;28.0]	11.1 [9.18;13.2]	
Gender (female)	148 (50.7%)	101 (52.6%)	47 (47%)	0.432
Age, years	60.0 [51.8; 68.0]	60.0 [50.8; 68.0]	59.5 [52.8; 67.0]	0.683
Retinopathy	145 (49.7%)	88 (45.8%)	57 (57.0%)	0.091
Education				0.672
Not even primary	38 (13.0%)	22 (11.5%)	16 (16.0%)	
Complete primary	165 (56.5%)	111 (57.8%)	54 (54.0%)	
Complete secondary	68 (23.3%)	44 (22.9%)	24 (24.0%)	
Graduate or higher	21 (7.19%)	15 (7.81%)	6 (6.0%)	
Caucasian	281 (96.2%)	189 (98.4%)	92 (92.0%)	0.009
Smoking (Current/Former/Never)	60/93/137	39/62/89	21/31/48	0.961
Diabetes duration (years)	8 [4.0; 15.0]	8 [4.0; 14.0]	10 [5.0; 15.0]	0.322
HbA1c (mmol/mol)	59.6 [51.4; 69.4]	56.8 [48.9; 66.1]	61.7 [51.9; 70.5]	0.049
Hypertension	165 (56.5%)	108 (56.2%)	57 (57.0%)	1.000
Dyslipidaemia	128 (43.8%)	88 (45.8%)	40 (40.0%)	0.407
Antiplatelet agents	114 (38.7%)	70 (36.5%)	43 (43.0%)	0.336
Psychotropic drugs	81 (27.7%)	50 (26.0%)	31 (31.0%)	0.447
Serum creatinine (mg/dl)	0.79 [0.68; 0.93]	0.80 [0.69; 0.92]	0.78 [0.66; 0.94]	0.561
Systolic BP (mmHg)	139 (18.7)	138 (18.3)	142 (19.1)	0.086
Diastolic BP (mmHg)	76.7 (10.7)	76.8 (10.6)	76.5 (11.0)	0.808
Waist (cm)	106 (11.8)	105 (11.0)	107 (13.1)	0.162
Diabetes treatment				0.489
Oral antidiabetic agents	158 (54.1%)	106 (55.2%)	52 (52.0%)	
Oral antidiabetic agents + Insulin	74 (25.3%)	45 (23.4%)	29 (29.0%)	
Insulin	22 (7.53%)	13 (6.77%)	9 (9.0%)	
Diet	38 (13.0%)	28 (14.6%)	10 (10.0%)	
Physical activity (> 25 min/day)	113 (38.7%)	68 (35.4%)	45 (45.0%)	0.142
Present quality of life	0.69 (1.15)	0.79 (1.09)	0.50 (1.24)	0.044
Diabetes-specific quality of life	−1.0 [−2.0; 0.0]	0.0 [−1.0; 0.0]	−1.0 [−2.0; 0.0]	0.001
Average weighted impact: ADDQoL score	0.58 [−1.23; −0.17]	−0.53 [− 1.08; − 0.17]	0.74 [− 1.59; − 0.18]	0.091
Perceived hyperglycaemia frequency	3.0 [1.0; 5.0]	3.0 [1.0; 5.0]	4.0 [1.0; 5.0]	0.257
Perceived hypoglycaemia frequency	0.0 [0.0; 2.0]	0.0 [0.0; 2.0]	0.0 [0.0; 2.0]	0.850
Final DTSQ score	27.0 [23.0; 30.0]	28.0 [23.0; 31.0]	25.0 [21.0; 28.2]	0.001

Values are shown as the mean (SD), median [interquartile range] or frequency (%). The *p*-values correspond to the unadjusted univariate analysis, which compares the difference for each variable between patients with and without vitamin D deficiency.

*HbA1c* glycated haemoglobin, *ADDQoL* Audit of Diabetes-Dependent Quality of Life, *DTSQ* Diabetes Treatment Satisfaction Questionnaire

## Results

Table 1 summarises the ADDQoL-19 and DTSQ-s results, clinical and socio-demographic characteristics, and their comparisons between the two study groups. Vitamin D concentrations were 22.7 [18.7; 28.0] ng/mL in patients without deficiency and 11.1 [9.18; 13.2] ng/mL in patients with vitamin D deficiency.

Unadjusted analysis in Table 2 shows that when vitamin D was used as an explanatory variable, the diabetes-specific quality of life score was significantly associated with a proportional increase in vitamin D concentration. However, when we used two groups of vitamin D levels (deficiency, < 15 ng/mL vs. non-deficiency > 15 ng/mL), the coefficients showed the differences between the means of both groups. These analysis, showed stronger associations of the study variables with vitamin D deficiency (< 15 ng/mL) than with serum vitamin D concentrations as a continuous variable. Vitamin D deficiency was significantly associated with lower quality of life according to all the measures of ADDQoL (average score (*p* = 0.020), present quality of life (*p* = 0.040) and diabetes-specific quality of life (*p* = 0.001), as well as with lower satisfaction with diabetes treatment (*p* = 0.004). The perception of hyperglycaemia or hypoglycaemia frequency did not show any significant association with vitamin D deficiency (*p* = 0.240 and *p* = 0.890, respectively).

**Table 2:** Unadjusted models of the association of vitamin D with quality of life and treatment satisfaction scores

	Vitamin D concentration^a^			Vitamin D deficiency^b^		
	Coefficient	SE	p	Coefficient	SE	p
Quality of life variables						
Average weighted impact: ADDQoL score	0.005	−0.007	0.430	−0.301	− 0.127	0.020
Present quality of life	0.007	−0.007	0.370	−0.292	−0.141	0.040
Diabetes-specific quality of life	0.016	−0.006	0.006	−0.393	−0.113	0.001
Treatment satisfaction variables						
Final score of DTSQ	0.064	−0.040	0.110	−2.212	−0.759	0.004
Perceived hyperglycaemias frequency	−0.015	0.014	0.320	0.333	−0.280	0.240
Perceived hypoglycaemias frequency	0.018	−0.012	0.130	0.034	−0.235	0.890

Regression coefficient (together with its standard error and p-value) estimated by simple linear regression models associated to vitamin D (25(OH) D serum concentration^a^, or the presence of vitamin D deficiency defined as levels of 25(OH) D < 15 ng/mL (37.4 nmol/L)^b^. Dependent variables are each of the three independent scores from each of the questionnaires, ADDQoL and DTSQ

As reported in our previous study [2], the multivariable analysis showed a second-order interaction between diabetes duration, the presence of diabetic retinopathy, and insulin therapy (*p* = 0.003). Diabetes-specific quality of life was significantly associated with three factors that interacted with each other: diabetes duration, treatment with insulin, and the presence or absence of diabetic retinopathy. Additionally, it was the only ADDQoL outcome keeping the significant association with vitamin D deficiency, showing a lower diabetic-specific quality of life in the multivariable regression model (*p* = 0.007) (Table 3).

**Table 3:** Multivariable linear model for diabetes-specific quality of life

Coefficient	Estimate	SE	*p*-value
Intercept	−0.214	0.171	0.218
Vitamin D deficiency^b^	−0.283	0.104	0.007
Diabetes duration (years)	−0.041	0.039	0.297
Diabetes duration-squared (years^2^)	0.002	0.002	0.271
Retinopathy	0.299	0.346	0.389
Insulin	1.409	0.793	0.077
Retinopathy^a^ insulin	−2.771	0.918	0.003
Diabetes duration^a^ insulin	−0.588	0.234	0.013
Diabetes duration-squared^a^ insulin	0.033	0.014	0.022
Diabetes duration^a^ retinopathy	−0.112	0.079	0.161
Diabetes duration-squared^a^ retinopathy	0.003	0.004	0.428
Diabetes duration^a^ retinopathy^a^ insulin	0.743	0.247	0.003
Diabetes duration-squared^a^ retinopathy^a^ insulin	−0.038	0.015	0.011

Multiple R-squared: 24.97%. ^a^denotes the existence of interactions between the variables. ^b^Vitamin D deficiency is defined as levels of 25(OH) D below 15 ng/mL (37.4 nmol/L)

Vitamin D deficiency was also significantly associated with a lower overall treatment satisfaction as assessed by the DTSQ (*p* = 0.020). As in the prior model [2], diabetic retinopathy had lower treatment satisfaction in relation to the duration of diabetes (*p* = 0.014) Moreover, former smokers had lower satisfaction compared with the group of non-smokers (*p* = 0.043). However, physically active patients had greater treatment satisfaction (*p* = 0.003) (Table 4).

**Table 4:** Multivariable linear model for final Diabetes Treatment Satisfaction Questionnaire score

Coefficient	Estimate	Standard error	*p*-value
Intercept	25.382	1.039	< 0.001
Vitamin D deficiency^b^	−1.734	0.740	0.020
Insulin	−1.500	0.961	0.119
Physical activity > 25 min	2.173	0.723	0.003
Current smoker	0.548	0.936	0.559
Former smoker	−1.628	0.803	0.043
Diabetes duration (years)	0.156	0.091	0.085
Retinopathy	1.954	1.191	0.102
Diabetes duration^a^ retinopathy	−0.265	0.107	0.014

Multiple R-squared: 12.78%. ^a^denotes the existence of interactions between the variables. ^b^Vitamin D deficiency is defined as levels of 25(OH) D below 15 ng/mL (37.4 nmol/L)

### Sensitivity analysis

Since some deviations from the normal distribution were observed in both multivariable linear models for extreme score values, and linear regression is highly influenced by them, the same models were fitted for the median scores by using quantile regression model, which is the robust alternative to linear regression to predict the median difference instead of the mean. The estimated adjusted coefficient to vitamin D deficiency (levels < 15 ng/dL) was − 0.491 in diabetes-specific quality of life, with a 95%CI of − 0.971 and − 0.166, showing also a significant reduction, even more significant than the one estimated of − 0.283 by applying multivariable linear regression. For diabetes treatment satisfaction score, the estimated adjusted coefficient of vitamin D deficiency was − 1.76, with a 95%CI of − 3.24 and − 0.64, showing also a significant reduction, very close to the − 1.73 value estimated by applying multivariable linear regression. Therefore, there is a significant reduction in both, diabetes-specific quality of life and diabetes treatment satisfaction associated with vitamin D deficiency.

## Discussion

The results of this study showed an association of vitamin D deficiency with decreased treatment satisfaction in patients with T2DM. Concerning HRQoL, although in global terms the average weighted impact according to the ADDQoL score was not associated with hypovitaminosis D, the results for the specific item that evaluated diabetes-related quality of life were significantly poorer in patients with vitamin D deficiency. To our knowledge, this is the first study that demonstrates the association of vitamin D deficiency with these important patient-reported outcomes in patients with type 2 diabetes mellitus.

The association found in the present study between vitamin D deficiency and quality of life in patients with T2DM is consistent with findings in other conditions. Specifically, in cross-sectional studies, an association has been reported between vitamin D deficiency (defined as 25 (OH) D < 20 ng/mL) and quality of life, assessed with specific questionnaires, in elderly Japanese women with osteoporosis who presented other comorbidities [13]. In Korean subjects with osteoarthritis, an independent association was also observed between vitamin D deficiency (25 (OH) D < 10 ng/mL) and a worse quality of life, as assessed by the EuroQOL-5 dimension, the EQ-5D index, and the EuroQOL-visual analogue scale (EQ-VAS) [14]. In a recent meta-analysis that mainly included intervention studies (73%), Hoffman et al. confirmed the weak to moderate benefits, in terms of self-reported quality of life, of vitamin D supplementation used on a short-term basis (< 6 months) in diseased populations (haemodialysis, rheumatic disease, heart failure, diffuse musculoskeletal pain or fatigue, sickle cell disease, chronic pain and Crohn’s disease) [30].

Concerning previous studies in diabetic patients, Krul-Poel et al. recently reported the results of a cross-sectional study that showed the absence of an association between vitamin D levels and HRQoL in non-vitamin D deficient Dutch subjects with T2DM [21]. Similarly, in an intervention study, the same research group could not show any improvements after six months of vitamin D supplementation (cholecalciferol 50,000 IU/month versus placebo) in patients who had T2DM with considerable associated comorbidities (micro- and macrovascular complications) [22]. In another intervention trial, Mager et al. reported that both daily (2000 IU/D) and monthly (40,000 IU/month) supplementation with vitamin D_3_ only correlated to a slight increase in the scores of the health-related quality of life questionnaire Short Form 36 Health Survey in elderly Canadian participants (95% with T2DM) with long-term diabetes duration (7–20 years) and chronic kidney disease [23]. At this point, it is very important to note that all these studies excluded subjects with vitamin D deficiency. Therefore, the previous evidence showing the lack of association of vitamin D or its supplementation with quality of life did not include subjects with vitamin D deficiency. Thus, our study is the first to address the issue of the association of vitamin D and QoL in subjects with type 2 diabetes without excluding those with lower concentrations of vitamin D.

The assessment of HRQoL through generic tools may also explain, at least partially, the differences reported. It should be highlighted that previous studies did not measure HRQoL using questionnaires specifically designed and validated for patients with diabetes. It is still common in the literature on chronic diseases to empirically assess HRQoL through the use of generic instruments [30]. The results of our research confirmed that, when specific questionnaires are used to assess quality of life, particularly those designed for the particular disease under consideration, different results may be obtained.

Despite treatment satisfaction as a subjective outcome measure in healthcare has been investigated in the past decades [31], this is the first study that shows a relationship between vitamin D status and treatment satisfaction in diabetic patients. We believe that it is very relevant to identify the factors that influence satisfaction with treatment in patients with chronic diseases, especially in diabetes. This outcome measure is considered an important indicator of the quality of healthcare, besides being a reliable indicator of adherence to treatment [3].

To the best of our knowledge, this is the first report of a positive association between vitamin D deficiency (< 15 ng/mL) and both diabetes-related quality of life and satisfaction with treatment in patients with T2DM non-supplemented with vitamin D. Additionally, this is the first study that used questionnaires that are specific for diabetes patients. The limitations of the present study include those that are intrinsic to its design. The use of a cross-sectional study design does not allow the establishment of a causal relationship between vitamin D deficiency and the study outcomes. Regarding the methodology, we acknowledge that the measurement of vitamin D concentrations was not done using liquid chromatography, the gold standard method for determination of vitamin D. However, we used a method that has been validated in other clinical studies. Additionally, it is important to note that this study was not primarily designed to assess the association between vitamin D deficiency and either HRQoL or treatment satisfaction. The absence of other advanced complications of diabetes in the participants of this study does not allow the extrapolation of the results to the general T2DM population. However, the general characteristics of study subjects are close to the general type 2 diabetes population in our region [32].

The question of whether vitamin D supplementation improves HRQoL and/or treatment satisfaction cannot be addressed with a study like the current one. Additionally, we cannot rule out the potential existence of a reverse association that may point to the fact that the HRQoL status or treatment satisfaction could be associated with behaviors that predispose patients to lower vitamin D levels.

## Conclusion

The current study showed that in patients with T2DM vitamin D deficiency is associated with a poorer perception of diabetes-specific quality of life and less satisfaction with diabetes treatment. Additionally, this research demonstrates the need to undertake further prospective and intervention studies to establish the role of the treatment of vitamin D deficiency in modifying the subjective measures of health status that are more important for patients with diabetes (HRQoL and satisfaction with treatment) and to determine the causal relationship between these variables.

## Abbreviations

ADDQoL: Audit of Diabetes Dependent Quality of Life
DTSQ: Diabetes Treatment Satisfaction Questionnaire
EQ-VAS: EuroQoL-visual analogue scale
HRQoL: Health-Related Quality of Life
T2DM: Type 2 diabetes mellitus
